# Survival after hematopoietic cell transplantation in children and adults in four decades of experience: nationwide study

**DOI:** 10.3389/fimmu.2026.1934596

**Published:** 2026-09-16

**Authors:** Małgorzata Dudkiewicz, Anna Łęczycka, Małgorzata Sobczyk-Kruszelnicka, Grzegorz Helbig, Kamil Wieczorek, Jan Kulkowski, Sebastian Giebel, Tomasz Czerw, Jan Maciej Zaucha, Andrzej Hellmann, Dorota Zielińska, Lidia Gil, Agnieszka Nowak-Potoczek, Agnieszka Wołoszyn, Patrycja Mensah-Glanowska, Joanna Skórzyńska, Tomasz Wróbel, Anna Czyż, Paula Jabłonowska-Babij, Krzysztof Kałwak, Marek Ussowicz, Agnieszka Cieniawa, Jarosław Dybko, Natalia Winciorek, Joanna Romejko-Jarosińska, Kazimierz Hałaburda, Jeremi Szypnicki, Piotr Rzepecki, Anna Owczarek, Agnieszka Wierzbowska, Olga Grzybowska-Izydorczyk, Grzegorz Basak, Iwona Marszałek, Jacek Wachowiak, Katarzyna Derwich, Agnieszka Sobkowiak-Sobierajska, Marek Hus, Magdalena Kozioł, Robert Dębski, Krzysztof Czyżewski, Jolanta Goździk, Agnieszka Dłużniewska, Jacek Krzanowski, Jarosław Kopiczak, Wojciech Legieć, Adrianna Wieluńska, Katarzyna Drabko, Teresa Greczkowska-Chmiel, Bogusław Machaliński, Sławomir Milczarek, Wojciech Homenda, Rafał Moraczyński, Grzegorz Charliński, Violetta Kalinowska, Iwona Malinowska, Michał Romiszewski, Katarzyna Brzeźniakiewicz-Janus, Katarzyna Koper, Mirosław Markiewicz, Rafał Peszuk, Marcin Pasiarski, Jarosław Czerwiński, Artur Kamiński, Wiesław Wiktor Jędrzejczak, Jan Styczyński

**Affiliations:** 1 Polish Transplant Coordinating Center Poltransplant CBMDR, Warsaw, Poland; 2 Department of Biochemistry and Microbiology, Warsaw University of Life Sciences, Warsaw, Poland; 3 Department of Bone Marrow Transplantation and Onco-Hematology, Maria Sklodowska-Curie National Research Institute of Oncology, Gliwice, Poland; 4 Department of Hematology and Bone Marrow Transplantation, Medical University of Silesia, Katowice, Poland; 5 Department of Hematology, Transplantology and Cell Therapies, Medical University of Gdańsk and University Clinical Center, Gdańsk, Poland; 6 Department of Hematology, Poznan University of Medical Sciences, Poznan, Poland; 7 Department of Hematology, University Hospital Kraków, Kraków, Poland; 8 Department of Hematology, Cellular Therapies and Internal Medicine, Wrocław Medical University, Wroclaw, Poland; 9 Department of Nursing, Faculty of Nursing and Midwifery, Wroclaw Medical University, Wroclaw, Poland; 10 Department of Pediatric Hematology/Oncology and BMT, Wrocław Medical University, Wroclaw, Poland; 11 Lower Silesia Centre of Oncology, Pulmonology, and Hematology; Department of Oncology and Hematology, Wroclaw University of Science and Technology, Wrocław, Poland; 12 Department of Lymphoid Malignancies, Maria Sklodowska-Curie National Research Institute of Oncology, Warsaw, Poland; 13 Institute of Hematology and Transfusion Medicine, Warsaw, Poland; 14 Department of Internal Medicine and Hematology Military Institute of Medicine - National Research Institute, Warsaw, Poland; 15 Department of Hematology, Medical University, Łódź, Poland; 16 Department of Hematology, Transplantation and Internal Medicine, Medical University of Warsaw, Warsaw, Poland; 17 Department of Pediatric Oncology, Hematology and Transplantology, Poznan University of Medical Sciences, Poznań, Poland; 18 Department of Hematology and Transplantology, Medical University, Lublin, Poland; 19 Department of Pediatric Hematology, Oncology, Immunology and Transplantology; Collegium Medicum Nicolaus Copernicus University Torun, Bydgoszcz, Poland; 20 Transplantation Center University Children’s Hospital in Krakow. Department Clinical Immunology and Transplantation Medical College Jagiellonian University, Krakow, Poland; 21 Brzozow Oncology Center, Brzozów, Poland; 22 Department of Hematology and Bone Marrow Transplantation, St. John of Dukla Oncology Center, Lublin, Poland; 23 Department of Pediatric Hematology, Oncology and Transplantology Medical University of Lublin, Lublin, Poland; 24 Department of Hematology and Bone Marrow Transplantation, Pomeranian Medical University, Szczecin, Poland; 25 Department of Hematology and Bone Marrow Transplantology, Regional Hospital; Institute of Health Sciences, Pomeranian University of Słupsk, Słupsk, Poland; 26 Department of Hematology, Nicolaus Copernicus Hospital, Toruń, Poland; 27 Department of Oncology, Pediatric Hematology, Clinical Transplantology, Pediatrics and Rare Disorders, Medical University of Warsaw, Warsaw, Poland; 28 Department of Hematology, Oncology and Radiotherapy; University of Zielona Góra, Multi-Specialist Hospital in Gorzów Wielkopolski, Gorzów Wielkopolski, Poland; 29 Department of Hematology, Faculty of Medicine, University Clinical Hospital, Collegium Medicum, University of Rzeszów, Rzeszow, Poland; 30 Department of Hematology, Holy Cross Cancer Centre; Department of Immunology, Jan Kochanowski University, Kielce, Poland; 31 Jurasz University Hospital 1, Bydgoszcz, Poland

**Keywords:** adults, children, hematopoietic cell transplantation, long-term follow-up, survival

## Abstract

**Objective:**

The objective of this study was to provide a comprehensive nationwide analysis of the development of hematopoietic cell transplantation (HCT) in Poland in the years 1984-2024, including temporal trends in transplant activity, transplant and donor types, stem cell sources, and patient survival.

**Methods:**

Centers reported basic data on all HCTs performed from the beginning of their activity up to the end of 2024. Survival data were identified in national registry.

**Results:**

The total number of HCTs was 30,812, including 40.1% allo-HCTs and 59.9% auto-HCTs. Pediatric centers performed 22.8% of all transplants; including 5.8% all auto-HCTs and 23.3% all allo-HCTs. Overall survival for children after allo-HCT was: 70.1% at 5 years and 67.2% at 10 years, while after auto-HCT was 57.1% at 5 years and 49.0% at 10 years. Overall survival for adults after allo-HCT was: 55.1% at 5 years and 49.0% at 10 years, while after auto-HCT was 73.5% at 5 years and 64.5% at 10 years. 10-years overall survival for children after allo-HCT with respect to donor type was 70.5% for MSD-HCT; 67.2% for UD-HCT; 44.8% for haplo-HCT; while for adults 49.5% for MSD-HCT; 49.6% for UD-HCT; 38.8% for haplo-HCT. Higher survival rates were observed in children <10 vs >10 years, and in adults <25 years vs >25 years. 10-years overall survival after HCT improved with subsequent time periods both in children and adults.

**Conclusions:**

Over a period of 40 years, increasing survival rates of allogeneic and autologous HCT are observed. There is an advantage in survival of younger patients.

## Introduction

Hematopoietic cell transplantation (HCT) is an effective treatment for numerous malignant and non-malignant diseases. Nevertheless, the high post-transplant mortality rate is influenced by several factors, including organ toxicity, graft-versus-host disease (GvHD), and infections (1–3). Since the first successful transplants, these methods have been continuously refined over the past 50 years, leading to a decline in mortality (1).

The first bone marrow transplant in history was performed by a Polish physician, Dr. Jan Raszek-Rosenbusch, who carried out the first allogeneic transplant in Lvov in 1938 on a child suffering from severe anemia. However, due to the Second World War, news of this achievement was not published until several years later (4).

In Europe, the number of transplants has risen significantly since the European Society for Blood and Marrow Transplantation (EBMT) began systematically collecting data. This growth has been accompanied by major changes in transplant practice, such as the use of peripheral blood stem cells, the establishment of donor registries, and improvements in HLA matching. During the 1990s, transplant rates in Eastern European countries remained lower than in Western Europe due to a limited number of transplant centers and more restricted access to suitable donors (5). The introduction of reduced-intensity conditioning (RIC) regimens and haploidentical donor transplants has significantly expanded the pool of patients eligible for allogeneic transplantation (6, 7).

Poland serves as a significant example of this transformation, evolving from a small number of pioneering procedures into a nationwide network of specialized transplant centers offering both autologous and allogeneic hematopoietic cell transplantation (HCT). The first successful HCT procedure in Poland was performed in 1984, marking the beginning of the development of hematopoietic cell transplantation in the country (8). Regular transplant activity in centers for adults began in 1985, and pediatric transplantation programs were launched in 1989 (9).

In subsequent years, the number of transplantation procedures gradually increased, accompanied by the establishment of new transplant centers. A significant milestone was reached in 1997, when the first hematopoietic cell transplantation from matched unrelated donor was performed in Poland (9). This achievement expanded donor availability for patients lacking a suitable family donor and contributed to the further development of allogeneic transplantation. Consequently, the hematopoietic cell transplantation procedure in Poland evolved from a pioneering intervention performed at a limited number of specialized facilities into an established treatment method available through a nationwide network of transplant centers. A timeline on **Figure 1** highlights the major milestones of HCT development in Poland, including the establishment of transplant programs, the first unrelated donor transplant, the development of national donor registries, and the introduction of haploidentical transplantation.

**Figure 1:**
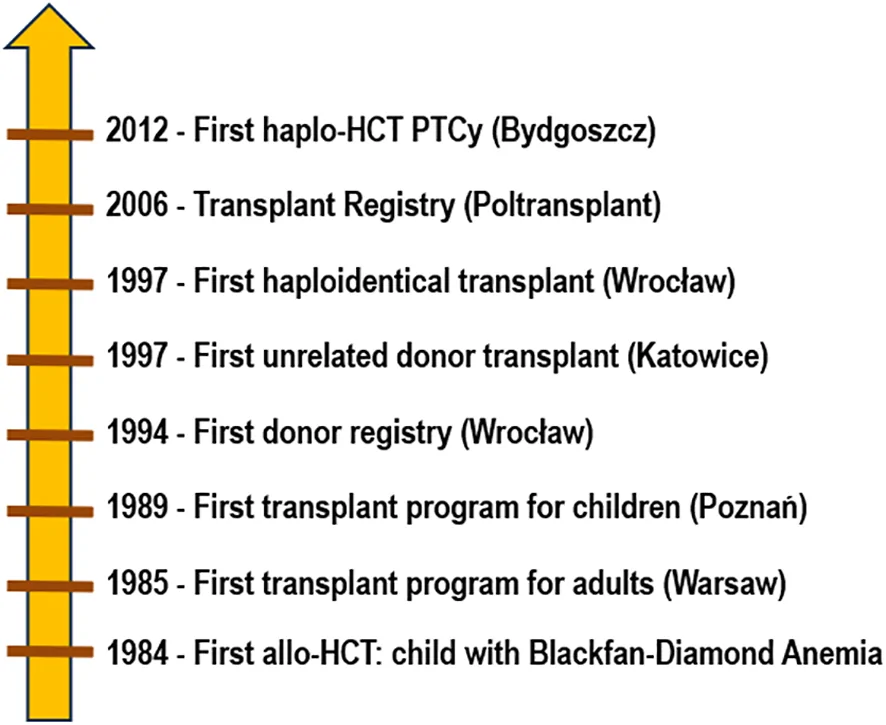
A timeline figure highlighting the major milestones of HCT development in Poland, including the establishment of transplant programs, the first unrelated donor transplant, the development of national donor registries, and the introduction of haploidentical transplantation.

In this population-based study, we demonstrate that advancements in standards of care for transplant patients over the past four decades have translated into improved overall survival (OS) in Poland. We analyzed data on all consecutive patients undergoing HCT in Poland and evaluated overall survival across specific patient subgroups. The study aimed to provide a comprehensive, nationwide analysis of the development of hematopoietic cell transplantation in Poland, accounting for changes over time regarding the number and type of transplants performed, donor types, stem cell sources, and patient survival.

## Methods

### Design

The study is a retrospective analysis of all hematopoietic cell transplants performed in Poland between 1984 and 2004. All consecutive transplants were included. All centers reported transplantation data, from the first procedure performed at a given center up to December 31, 2024.

### Patients

The following data were collected at transplant centers: date of birth, date of transplantation (initial and subsequent, if performed), primary diagnosis, type of transplantation, donor type (in the case of allogeneic transplantation), stem cell source, date of last follow-up, and date of death (if applicable).

### Transplant centers

Located in Central Europe, Poland has a population of over 37 million and an area of ​​313,933 km². The country is divided into 16 administrative regions. Almost all regions have medical facilities that provide hematopoietic cell transplantation (HCT) care and submit data to the Polish National Transplant Registry (NTR) managed by Poltransplant (the Polish Coordination Centre for Transplantation, based in Warsaw).

All 27 medical centers performing HCT procedures participated in the study, including 21 centers for adults and 6 pediatric centers. None of the centers employed time intervals when performing HCT procedures. Transplant centers in Poland: Department of Hematology and Bone Marrow Transplantation, Medical University of Silesia in Katowice (period covered: 1992-2024); Department of Bone Marrow Transplantation and Onco-Hematology, Maria Sklodowska-Curie National Research Institute of Oncology in Gliwice (period covered: 2010-2024); Department of Hematology, Transplantology and Cell Therapies, Medical University of Gdańsk and University Clinical Center in Gdańsk (period covered: 1994-2024); Department of Hematology, Poznan University of Medical Sciences in Poznan (period covered: 1990-2024); Department of Hematology, University Hospital in Kraków (period covered: 1998-2024); Department of Hematology, Cellular Therapies and Internal Medicine, Wrocław Medical University in Wroclaw (period covered: 2000-2024); Department of Pediatric Hematology/Oncology and BMT, Wrocław Medical University in Wroclaw (period covered: 1994-2024); Lower Silesia Centre of Oncology, Pulmonology, and Hematology; Department of Oncology and Hematology, Wroclaw University of Science and Technology in Wrocław (period covered: 1987-2024); Department of Lymphoid Malignancies, Maria Sklodowska-Curie National Research Institute of Oncology in Warsaw (period covered: 1997-2024); Institute of Hematology and Transfusion Medicine in Warsaw (period covered: 1999-2024); Department of Internal Medicine and Hematology Military Institute of Medicine - National Research Institute in Warsaw (period covered: 1984-2024); Department of Hematology, Medical University in Łódź (period covered: 2000-2024); Department of Hematology, Transplantation and Internal Medicine, Medical University in Warsaw (period covered: 1999-2024); Department of Pediatric Oncology, Hematology and Transplantology, Poznan University of Medical Sciences in Poznań (period covered: 1989-2024); Department of Hematology and Transplantology, Medical University in Lublin (period covered: 1997-2024); Department of Pediatric Hematology, Oncology, Immunology and Transplantology; Collegium Medicum Nicolaus Copernicus University and Jurasz University Hospital in Bydgoszcz (period covered: 2003-2024); Transplantation Center University Children’s Hospital in Krakow. Department Clinical Immunology and Transplantation Medical College Jagiellonian University in Krakow (period covered: 2002-2024), Oncology Center in Brzozów (period covered: 2017-2024); Department of Hematology and Bone Marrow Transplantation, St. John of Dukla Oncology Center in Lublin (period covered: 2023-2024); Department of Pediatric Hematology, Oncology and Transplantology Medical University in Lublin (period covered: 1998-2024); Department of Hematology and Bone Marrow Transplantation, Pomeranian Medical University in Szczecin (period covered: 2018-2024); Department of Hematology and Bone Marrow Transplantology, Regional Hospital; Institute of Health Sciences, Pomeranian University in Słupsk (period covered: 2020-2024); Department of Hematology, Nicolaus Copernicus Hospital in Toruń (period covered: 2017-2024); Department of Oncology, Pediatric Hematology, Clinical Transplantology, Pediatrics and Rare Disorders, Medical University in Warsaw (period covered: 2018-2024); Department of Hematology, Holy Cross Cancer Centre, Department of Immunology, Jan Kochanowski University in Kielce (period covered: 2015-2024); Department of Hematology, Oncology and Radiotherapy, University of Zielona Góra, Multi-Specialist Hospital in Gorzów Wielkopolski (period covered: 2024-2024); Department of Hematology, Faculty of Medicine, University Clinical Hospital, Collegium Medicum, University of Rzeszów in Rzeszow (period covered: 2024-2024).

### Registry platform

The National Transplant Registry (NTR) in Poland is an electronic system developed by the Poltransplant organization (10). Since 2006, all basic information on allogeneic transplants are collected in NTR, including pre-transplant patient and donor data, details on the stem cell source, and the date of transplantation.

The NTR system operates on most standard web browsers, eliminating the need for additional software installation, and features a user-friendly, ergonomic interface. It is equipped with built-in security mechanisms; access is restricted to authorized users assigned specific roles, and login requires secure credentials. Data entry is performed by authorized medical personnel (physicians, nurses) or designated hospital staff responsible for data management. Finally, all patient data undergo pseudonymization, and each patient is assigned a unique identifier.

### Collection and validation of data

Data from the NTR were used for this study; all other data regarding allo-HCT procedures performed prior to 2006 and data concerning auto-HCT were collected at individual centers using a form specifically designed for this purpose.

Transplantation data, including information on patients, donors (in the case of allogeneic transplants), transplant procedures, and available survival data, were collected by transplant coordinators at individual centers using a form specifically designed for this purpose. The collected data were submitted to Poltransplant, the Coordination and Organization Centre for Transplantation (comprising the Central Bone Marrow Donor Registry [CBMDR] and the NTR in Warsaw), where they were consolidated into a single database.

Missing survival data and unclear cases were verified Poltransplant using transplant recipient’s unique national identification numbers listed in PESEL system (Universal Electronic Population Registration System [Powszechny Elektroniczny System Ewidencji Ludności]). **Figure 2** presents a study flowchart showing the number of records initially identified, duplicates, exclusions, unique patients, first transplants, subsequent transplants, and the number of patients included in analyses.

**Figure 2:**
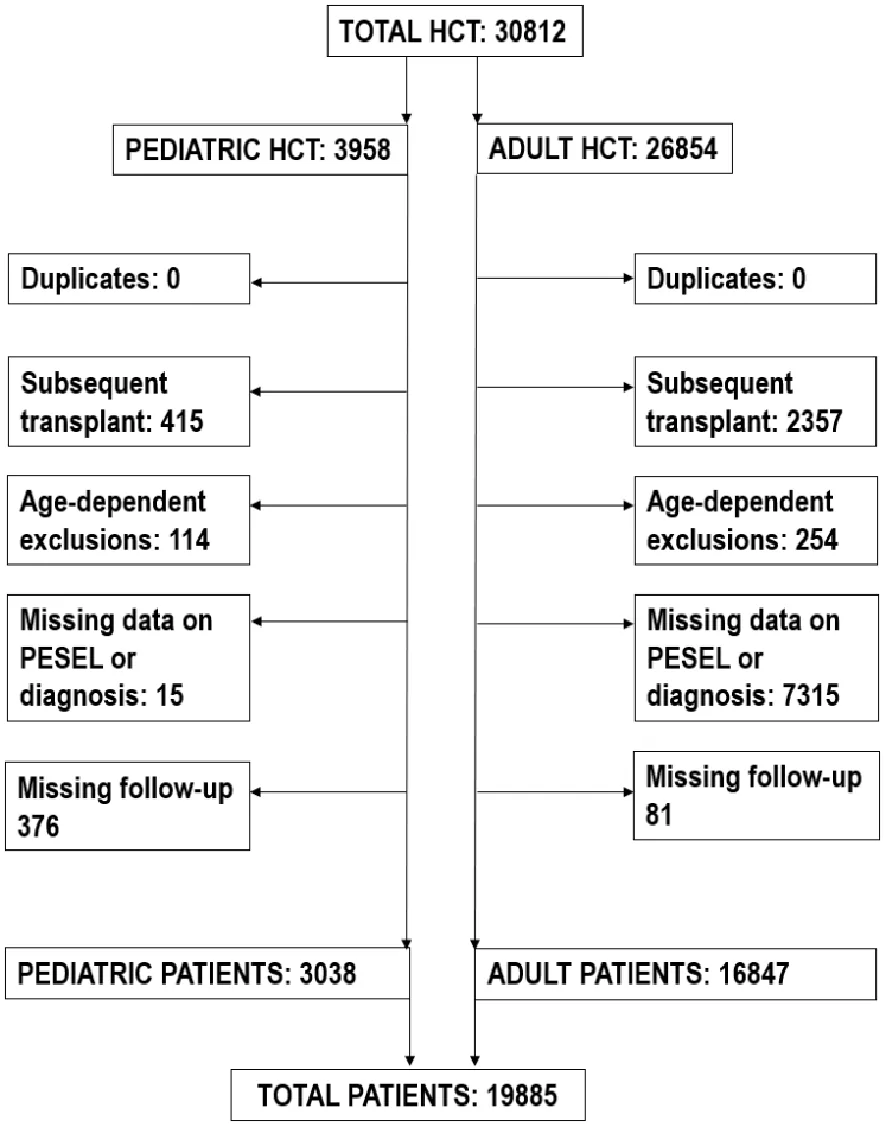
A study flowchart showing the number of records initially identified, duplicates, exclusions, unique patients, first transplants, subsequent transplants, and the number of patients included in analyses.

Dates of death were validated upon four additional data sources: checked against the central PESEL system, linked to the Registry of Objections. Data obtained from the centers was cross-referenced with entries in the NTR, the CBMDR database, and the registry of hematopoietic stem cells imports.

### Definitions

Given the varying standards and procedural techniques, as well as the long patient enrollment period, the following definitions were adopted: matched related donor (MRD) - a family donor who is HLA-matched or has a single HLA mismatch; unrelated donor (MUD/UD) - a non-family donor who is HLA-matched or has 1-2 HLA mismatches; haploidentical donor - any family donor with ≥2 HLA mismatches or an unrelated donor with >2 HLA mismatches. Due to the very small number of cord blood transplantations (CBT), these procedures were classified into the MRD, MUD, or haplo-HCT groups.

### Ethics statement

All investigations and therapeutic procedures constituted established clinical practices and were performed in accordance with accepted clinical guidelines and the requirements of the Declaration of Helsinki. In accordance with standard procedure, informed consent for treatment and data use was obtained from all patients or their parents or legal guardians prior to the commencement of treatment. No personal data were included in the study. Due to the retrospective nature of the study, which was based on registry data, approval from an ethics committee was not required.

### Data set

The data set remains the property of Poltransplant (The Polish Transplant Coordinating Center, Warsaw, Poland).

### Statistical analysis

Overall survival for each patient was calculated from the time of the first transplantation until death or the last follow-up visit. Death was treated as the event of interest, while loss to follow-up was treated as a censoring event. For patients alive at the time of their last available follow-up visit, data were censored at the date of that visit. Patients who underwent more than one transplantation procedure were analyzed for overall survival based on the type of the first transplantation, considering the period up to the last follow-up visit. Follow-up ended on December 31, 2024. The following factors were analyzed: type of transplantation (allogeneic vs. autologous), age group (pediatric vs. adult patients), type of donor (matched family vs unrelated vs family mismatched/haploidentical), and calendar period (1984–2000, 2000–2010, and 2010–2024). Survival curves were generated using the Kaplan-Meier method and compared using the log-rank test.

## Results

### Demographics for survival analysis

After applying all exclusion criteria (**Figure 2**), a total of 19,885 patients were included in the long-term survival analysis: 3,038 children and 16,847 adults (**Table 1**). The median follow-up time was 5.5 years for children undergoing allo-HCT and 5 years for those undergoing auto-HCT, and 4.1 years and 3.0 years for adults, respectively.

**Table 1:** Characteristics of patients and transplants.

Characteristics	Children	Adults	Total
Number of transplants	3958	26854	30812
First transplants	3414	16928	20342
Patients included in survival analysis	3038	16847	19885
Transplant period	3038 (100%)	16847 (100%)	19885 (100%)
1974-2000	255 (8.39%)	809 (4.80%)	1064 (5.35%)
2000-2010	1026 (33.77%)	3604 (21.39%)	4630 (23.28%)
2010-2024	1757 (57.83%)	12434 (73.81%)	14191 (71.37%)
HCT type	3038 (100%)	16847 (100%)	19885 (100%)
Autologous	858 (28.24%)	9569 (56.80%)	10427 (52.44%)
Allogeneic	2180 (71.76%)	7278 (43.20%)	9458 (47.56%)
Donor type	2180 (100%)	7278 (100%)	9458 (100%)
Sibling (MSD)	715 (32.80%)	2592 (35.61%)	3307 (34.97%)
Unrelated	1344 (61.65%)	4240 (58.26%)	5584 (59.04%)
Haplo/MMRD	121 (5.55%)	446 (6.13%)	567 (5.99%)
Stem cell source	3038 (100%)	16847 (100%)	19885 (100%)
Peripheral Blood	1856 (61.09%)	15419 (91.52%)	17275 (86.87%)
Bone Marrow	1087 (35.78%)	1311 (7.78%)	2398 (12.06%)
Cord Blood	32 (1.05%)	16 (0.09%)	48 (0.24%)
Combined	32 (1.05%)	101 (0.60%)	133 (0.67%)
Unknown	31 (1.02%)	0	31 (0.16%)

MSD, matched sibling donor; MMRD, mismatched related donor.

### Overall number of transplants

Between 1984 and 2024, hematopoietic cell transplantations in Poland were performed at a total of 27 transplant centers; 20 of these centers performed both allogeneic and autologous transplantations, while 7 centers performed only autologous transplantations (auto-HCT). A total of 30,812 transplantations were performed, including 12,359 (40.1%) allo-HCT and 18,453 (59.9%) auto-HCT procedures. Within the allo-HCT group, 7,044 (57.0%) procedures were performed from an unrelated donor (MUD or MMUD); 4,468 (36.3%) from a matched related donor (MRD); and 829 (6.7%) were haploidentical transplantations (haplo-HCT) (**Table 2**). The distribution of transplantation types according to donor and calendar year is shown in **Figures 3A, B**.

**Table 2:** Number of HCTs performed in Poland between 1984–2024.

Type of center	Total	AUTO-HCT	ALLO-HCT			
			Total allo	MRD	MUD	Haplo
Total (27 centers)	30812	18453 (59.9%)	12359 (40.1%)	4468 (14.5%)	7044 (22.9%)	829 (2.7%)
Adult (21 centers)	26854	17374 (64.7%)	9480 (35.3%)	3616 (13.5%)	5228 (19.5%)	618 (2.3%)
Pediatric (6 centers)	3958	1079 (27.3%)	2879 (72.7%)	852 (21.5%)	1816 (45.9%)	211 (5.3%)

HCT, hematopoietic cell transplantation; auto, autologous; allo, allogeneic; MRD, matched related donor; MUD, matched/mismatched unrelated donor; haplo, haploidentical.

**Figure 3:**
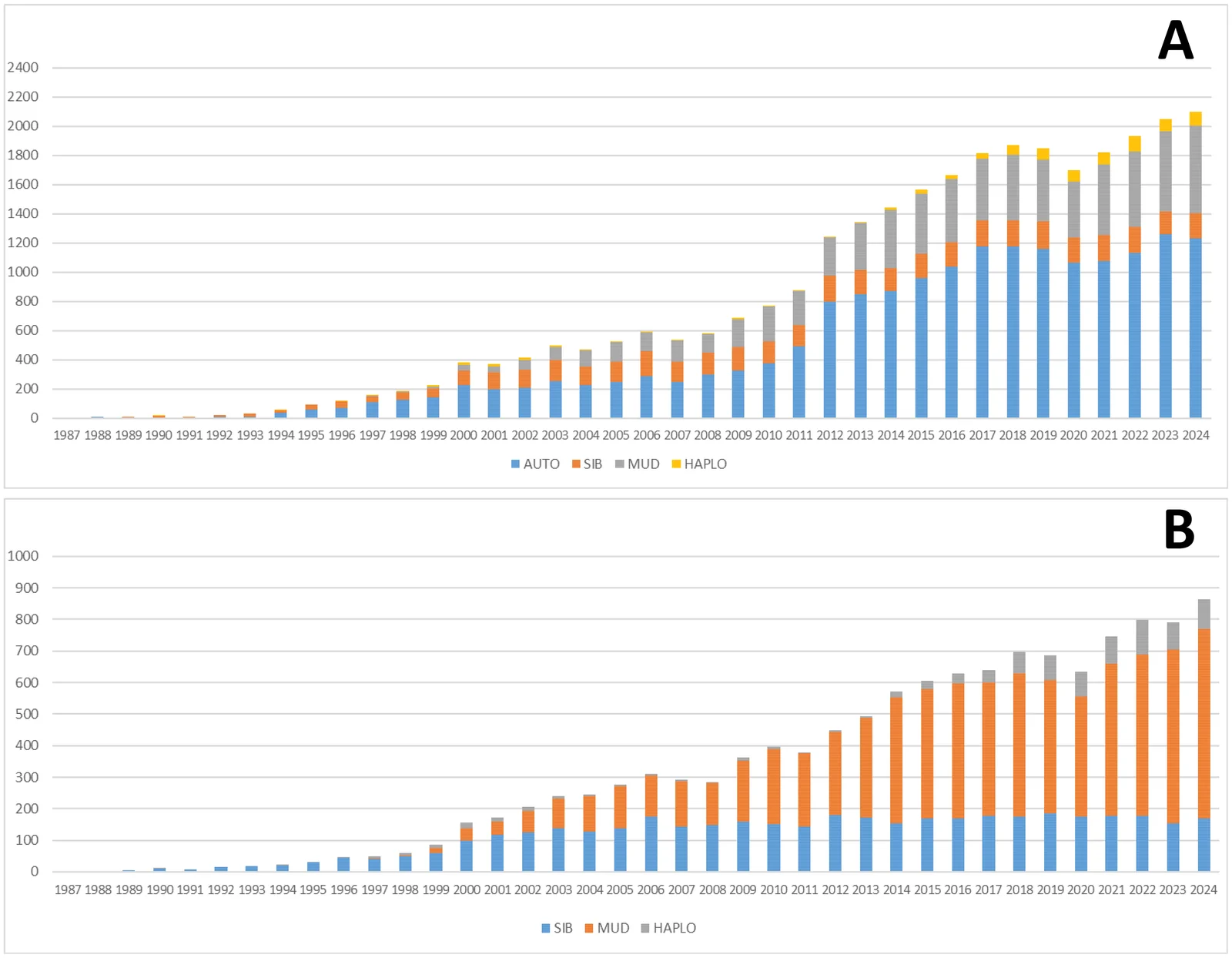
Overall number of autologous and allogeneic transplants in Poland between 1987-20024: **(A)** Overall; **(B)** Allogeneic only. HCT, hematopoietic cell transplantation; auto, autologous; allo, allogeneic; MRD, matched related donor; MUD, matched/mismatched unrelated donor; haplo, haploidentical.

Adult centers. Overall, total number of HCTs 26’773, including 9663 (36.1%) allo-HCT and 17110 (63.9%) auto-HCT. With respect to donor type, among allogeneic-HCT, 5339 (55.3%) MUD, 3669 (38.0%) MRD and 642 (6.7%) haplo-HCT were performed. Adult centers performed 77.2% of all transplants; 94.2% all auto- and 76.7% all allo-HCTs. Among allo-HCT, adult centers performed 80.9% all MRD, 74.2% all MUD and 74.5% all haplo-HCTs.

Pediatric centers. Overall, total number of HCTs was 3’958, including 2879 (72.7%) allo-HCT and 1079 (27.3%) auto-HCT. With respect to donor type, among allogeneic-HCT, 1816 (63.1%) MUD, 852 (29.6%) MRD and 211 (7.3%) haplo-HCT were performed. Pediatric centers performed 12.8% of all transplants; 5.8% all auto- and 23.3% all allo-HCTs. Among allo-HCT, pediatric centers performed 19.1% all MRD, 25.8% all MUD and 25.5% all haplo-HCTs.

### Primary indications for transplantation

Primary indications for transplantation varied between children and adults (**Table 3**).

**Table 3:** Primary indications for transplantation in children and adults.

Diagnosis	Children		Adults		Total		TOTAL
	Autologous	Allogeneic	Autologous	Allogeneic	Autologous	Allogeneic	
**HEMATOLOGICAL MALIGNANCIES**	**213 (24.83%)**	**1511 (69.31%)**	**9031 (94.38%)**	**6758 (92.86%)**	**9244 (88.65%)**	**8269 (87.43%)**	**17513 (88.07%)**
ALL (acute lymphoblastic leukemia)	31 (3.61%)	769 (35.28%)	172 (1.80%)	1210 (16.63%)	203 (1.95%)	1979 (20.92%)	2182 (10.97%)
AML (acute myeloid leukemia)	45 (5.24%)	398 (18.26%)	220 (2.30%)	3124 (42.92%)	265 (2.54%)	3522 (37.24%)	3787 (19.04%)
BAL (biphenotypic leukemia)	2 (0.23%)	17 (0.78%)	-	10 (0.14%)	2 (0.02%)	27 (0.29%)	29 (0.15%)
JMML (juvenile myelomonocytic leukemia)	-	13 (0.60%)	-	-	-	13 (0.14%)	13 (0.07%)
CML (chronic myeloid leukemia)	1 (0.12%)	92 (4.22%)	17 (0.18%)	623 (8.56%)	18 (0.17%)	715 (7.56%)	733 (3.69%)
CLL (chronic lymphocytic leukemia)	-	-	10 (0.10%)	125 (1.72%)	10 (0.10%)	125 (1.32%)	135 (0.68%)
Other leukemias	1 (0.12%)	3 (0.14%)	1 (0.01%)	87 (1.20%)	2 (0.02%)	90 (0.95%)	92 (0.46%)
MDS (myelodysplastic syndromes)	-	129 (5.92%)	5 (0.05%)	625 (8.59%)	5 (0.05%)	754 (7.97%)	759 (3.82%)
MPS/MPN (myeloproliferative neoplasms)	2 (0.23%)	1 (0.05%)	-	257 (3.53%)	2 (0.02%)	258 (2.73%)	260 (1.31%)
MM/MP (multiple myeloma/plasmocytoma)	-	-	4165 (43.53%)	118 (1.62%)	4165 (39.94%)	118 (1.25%)	4283 (21.54%)
HD (Hodgkin lymphoma)	65 (7.58%)	8 (0.37%)	1656 (17.31%)	233 (3.20%)	1721 (16.51%)	241 (2.55%)	1962 (9.87%)
NHL (non-Hodgkin lymphoma)	66 (7.69%)	81 (3.72%)	2785 (29.10%)	346 (4.75%)	2851 (27.34%)	427 (4.51%)	3278 (16.48%)
**SOLID TUMORS**	**639 (74.48%)**	**14 (0.64%)**	**293 (3.06%)**	**8 (0.11%)**	**932 (8.94%)**	**22 (0.23%)**	**954 (4.80%)**
Solid tumors (not otherwise specified)	295 (34.38%)	12 (0.55%)	292 (3.05%)	8 (0.11%)	587 (5.63%)	20 (0.21%)	607 (3.05%)
Neuroblastoma	223 (25.99%)	2 (0.09%)	1 (0.01%)	-	224 (2.15%)	2 (0.02%)	226 (1.14%)
Ewing sarcoma	59 (6.88%)	-	-	-	59 (0.57%)	-	59 (0.30%)
Germ cell tumor	17 (1.98%)	-	-	-	17 (0.16%)	-	17 (0.09%)
Nephroblastoma	9 (1.05%)	-	-	-	9 (0.09%)	-	9 (0.05%)
CNS tumors	36 (4.20%)	-	-	-	36 (0.35%)	-	36 (0.18%)
**NON-MALIGNANT DISEASES**	**5 (0.58%)**	**654 (30.00%)**	**246 (2.57%)**	**512 (7.03%)**	**251 (2.41%)**	**1166 (12.33%)**	**1417 (7.13%)**
SAA/AA + IBMF (bone marrow failures*)	1 (0.12%)	306 (14.04%)	-	285 (3.92%)	1 (0.01%)	591 (6.25%)	592 (2.98%)
Primary immunodeficiences	-	263 (12.06%)	2 (0.02%)	9 (0.12%)	2 (0.02%)	272 (2.88%)	274 (1.38%)
Autoimmune diseases	2 (0.23%)	3 (0.14%)	103 (1.08%)	6 (0.08%)	105 (1.01%)	9 (0.10%)	114 (0.57%)
Amyloidosis	-	-	22 (0.23%)	-	22 (0.21%)	-	22 (0.11%)
Metabolic diseases	-	62 (2.84%)	14 (0.15%)	-	14 (0.13%)	62 (0.66%)	76 (0.38%)
PNH (paroxysmal nocturnal hemoglobinuria)	-	3 (0.14%)	-	45 (0.62%)	-	48 (0.51%)	48 (0.24%)
Other	2 (0.23%)	17 (0.78%)	105 (1.10%)	167 (2.29%)	107 (1.03%)	184 (1.95%)	291 (1.46%)
**TOTAL NUMBER OF HCT BY PRIMARY DIAGNOSES**	**858 (100%)**	**2180 (100%)**	**9569 (100%)**	**7278 (100%)**	**10427 (100%)**	**9458 (100%)**	**19885 (100%)**
Subsequent HCTs or missing diagnosis data or missing PESEL/follow-up data	221	699	7805	2202	8026	2901	10927
Total number of HCTs	1079	2879	17374	9480	18453	12359	30812

*SAA/AA, (severe) aplastic anemia; IBMF, inborn bone marrow failure; FA, Fanconi Anemia; BDA, Blackfan-Diamond Anemia; SDS, Schwachman-Diamond Syndrome; DC, Congenital Dyskeratosis.

In bold: groups of diseases and total number of patients.

Children. Most often indication for allo-HCT were acute lymphoblastic leukemia (ALL; 35.28%), acute myeloid leukemia (AML; 18.26%) and myelodysplastic syndromes (MDS; 5.92%) among malignant diseases, while severe aplastic anemia/congenital bone marrow failures (SAA/AA/BMF; 14.04%) and primary immunodeficiences (inborn error of immunity; IEI; 12.06%) among non-malignant diseases. Most often indication for auto-HCT were neuroblastoma and other solid tumors (without specific diagnosis) in 60.37% of all auto-HCTs.

Adults. Most often indication for allo-HCT were AML (42.92%), ALL (16.63%), MDS (8.59%) and chronic myeloid leukemia (CML; 8.56%). Most often indications for auto-HCT were multiple myeloma (MM/MP; 43.53%), non-Hodgkin lymphoma (NHL; 29.10%), and Hodgkin disease/lymphoma (HD/HL; 17.31%).

### Overall survival in children (allogeneic vs autologous HCT)

Children. Overall survival for all children after allo-HCT (n=2180) was: 70.1% at 5 years and 67.2% at 10 years, while for all children after auto-HCT (n=858) was 57.1% at 5 years and 49.0% at 10 years (p<0.00001) (**Figure 4A**).

**Figure 4:**
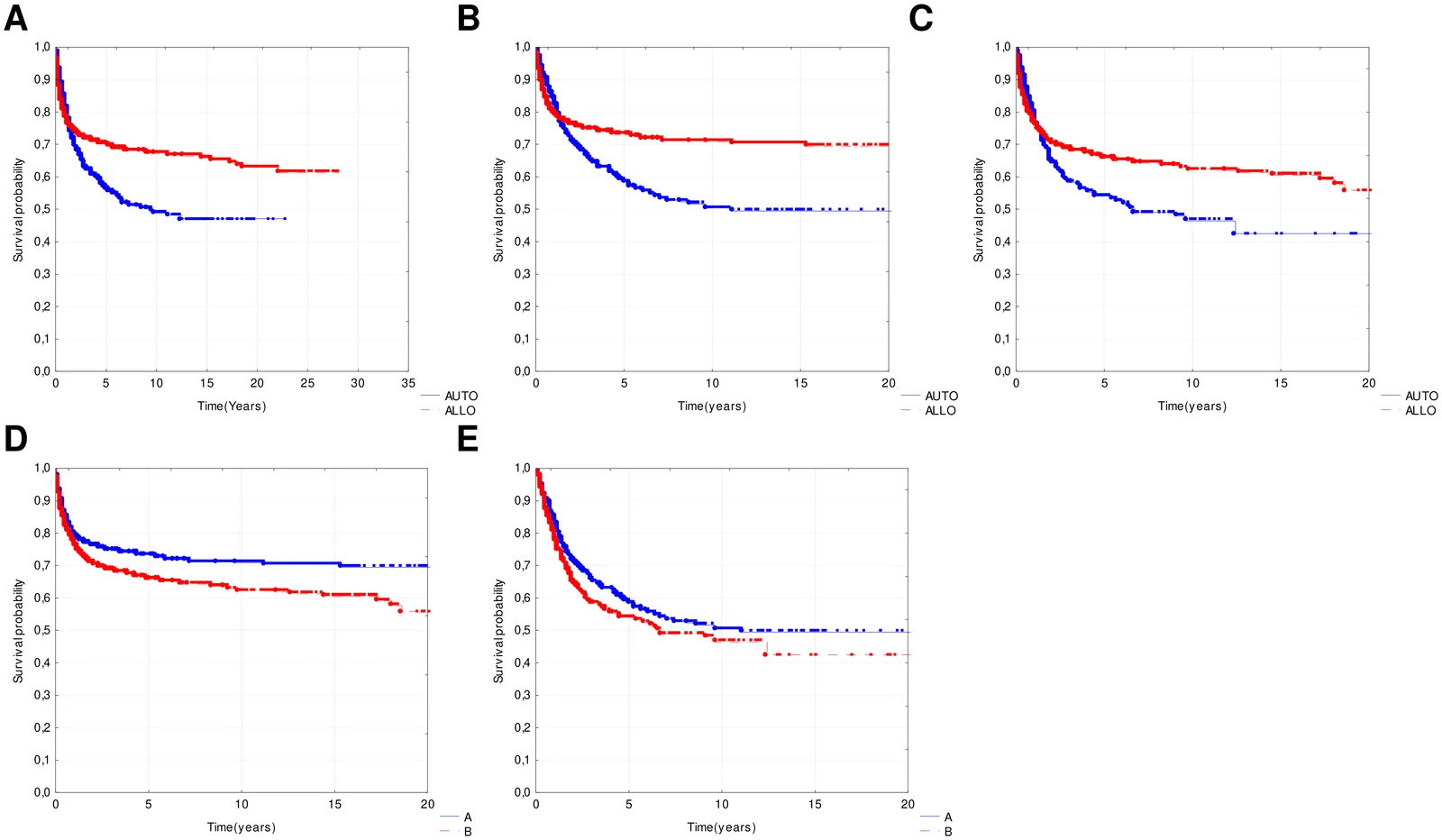
Overall survival after allo-HCT and auto-HCT in children: **(A)** total; **(B)** age <10 years; **(C)** age >10 years; **(D)** OS after allo-HCT in children <10 years vs >10 years; **(E)** OS after auto-HCT in children <10 years vs >10 years.

Children <10 years. Overall survival for children aged <10 years after allo-HCT (n=1207) was: 73.3% at 5 years and 70.8% at 10 years, while after auto-HCT (n=531) was 59.1% at 5 years and 50.6% at 10 years (p=0.00001) (**Figure 4B**).

Children >10 years. Overall survival for children aged >10 years after allo-HCT (n=973) was: 66.1% at 5 years and 62.5% at 10 years, while after auto-HCT (n=327) was 54.0% at 5 years and 46.4% at 10 years (p=0.00611) (**Figure 4C**).

Children after allo-HCT. Overall survival for children aged <10 years after allo-HCT (n=1207) was: 73.3% at 5 years and 70.8% at 10 years; while in children aged >10 years after allo-HCT (n=973) was: 66.1% at 5 years and 50.6% at 10 years (p=0.00033) (**Figure 4D**).

Children after auto-HCT. Overall survival for children aged <10 years after auto-HCT (n=531) was: 59.1% at 5 years and 50.7% at 10 years, while in children aged >10 years after auto-HCT (n=327) was 54.1% at 5 years and 46.4% at 10 years (p=0.06736) (**Figure 4E**).

### Overall survival in adults (allogeneic vs autologous HCT)

Adults. Overall survival for all adults after allo-HCT (n=7288) was: 55.1% at 5 years and 49.0% at 10 years, while for all adults after auto-HCT (n=9573) was 73.5% at 5 years and 64.5% at 10 years (p<0.00001) (**Figure 5A**).

**Figure 5:**
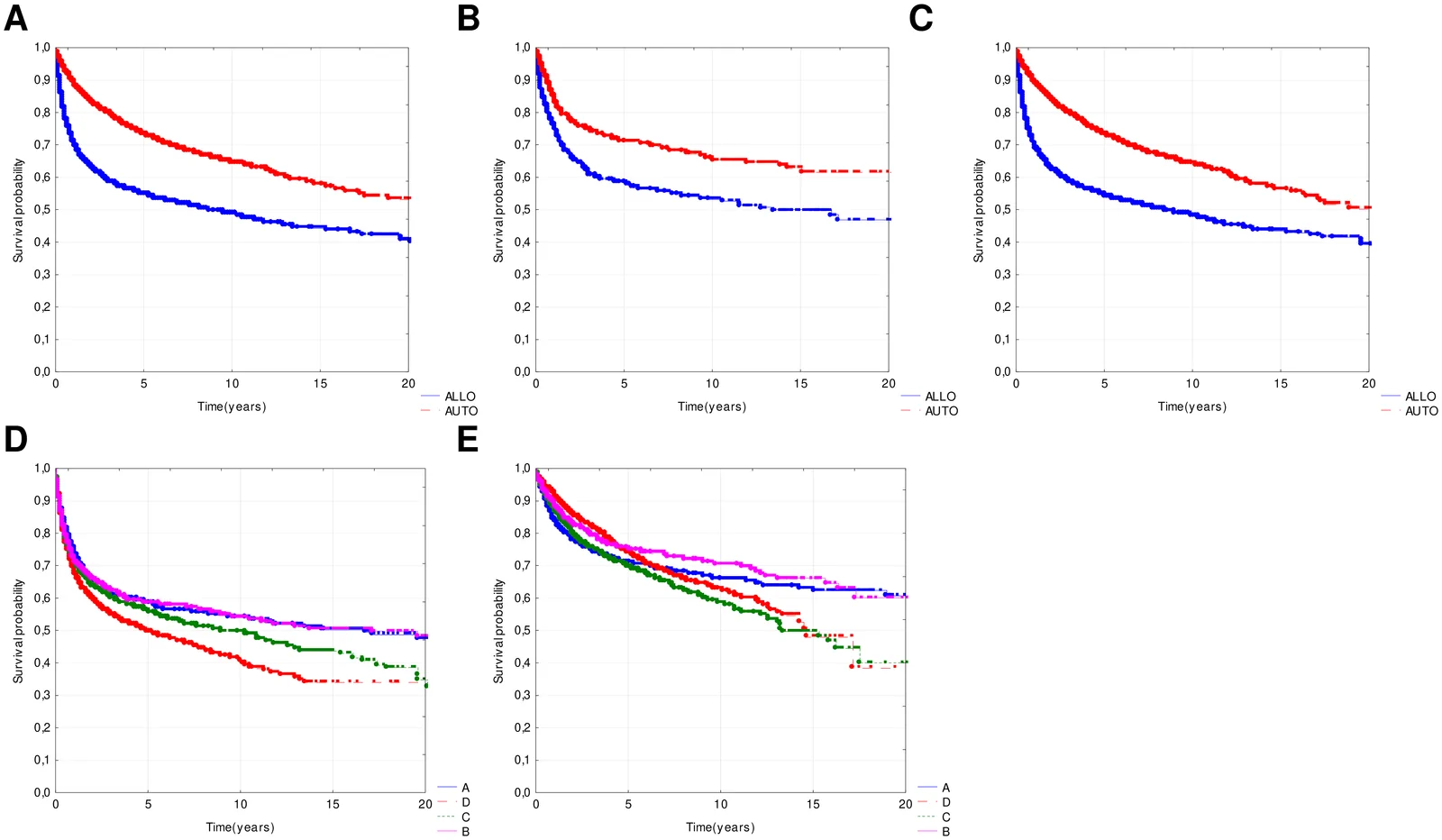
Overall survival after allo-HCT and auto-HCT in adults: **(A)** total; **(B)** age 18-25 years; **(C)** age >25 years; **(D)** OS after allo-HCT by age groups (age groups: A, 18-30y; B, 30-40y; C, 40-50y; D, >50y) in adults; **(E)** OS after auto-HCT by age groups in adults.

Adults 18-25 years. Overall survival for adults aged 18-25 years after allo-HCT (n=791) was: 58.3% at 5 years and 53.2% at 10 years, while after auto-HCT (n=675) was 71.1% at 5 years and 65.9% at 10 years (p<0.00001) (**Figure 5B**).

Adults >25 years. Overall survival for adults aged >25 years after allo-HCT (n=6497) was: 54.6% at 5 years and 48.4% at 10 years, while after auto-HCT (n=8898) was 73.5% at 5 years and 64.2% at 10 years (p<0.00001) (**Figure 5C**).

Adults after allo-HCT. Overall survival for adults aged 18-30 years after allo-HCT (n=1539) was: 59.4% at 5 years and 54.2% at 10 years; in patients aged 30-40 years (n=1364) was: 58.6% at 5 years and 53.9% at 10 years; in patients aged 40-50 years (n=1587) was: 56.1% at 5 years and 49.7% at 10 years; in patients aged >50 years (n=2798) was: 50.0% at 5 years and 40.9% at 10 years (p=0.00015) (**Figure 5D**).

Adults after auto-HCT. Overall survival for adults aged 18-30 years after auto-HCT (n=1414) was: 72.2% at 5 years and 66.1% at 10 years; in patients aged 30-40 years (n=1199) was: 75.3% at 5 years and 70.6% at 10 years; in patients aged 40-50 years (n=1647) was: 69.9% at 5 years and 58.3% at 10 years; in patients aged >50 years (n=5313) was: 74.3% at 5 years and 62.7% at 10 years (p<0.00001) (**Figure 5E**).

### Outcome after allo-HCT (MSD, UD, HAPLO) in children

Children. Overall survival for all children after allo-HCT (n=2180) with respect to donor type: for MSD-HCT (n=716) was 73.2% at 5 years and 70.5% at 10 years; for UD-HCT (n=1343) was 70.6% at 5 years and 67.2% at 10 years; for haplo-HCT (n=121) was 46.4% at 5 years and 44.8% at 10 years (p<0.00001) (**Figure 6A**).

**Figure 6:**
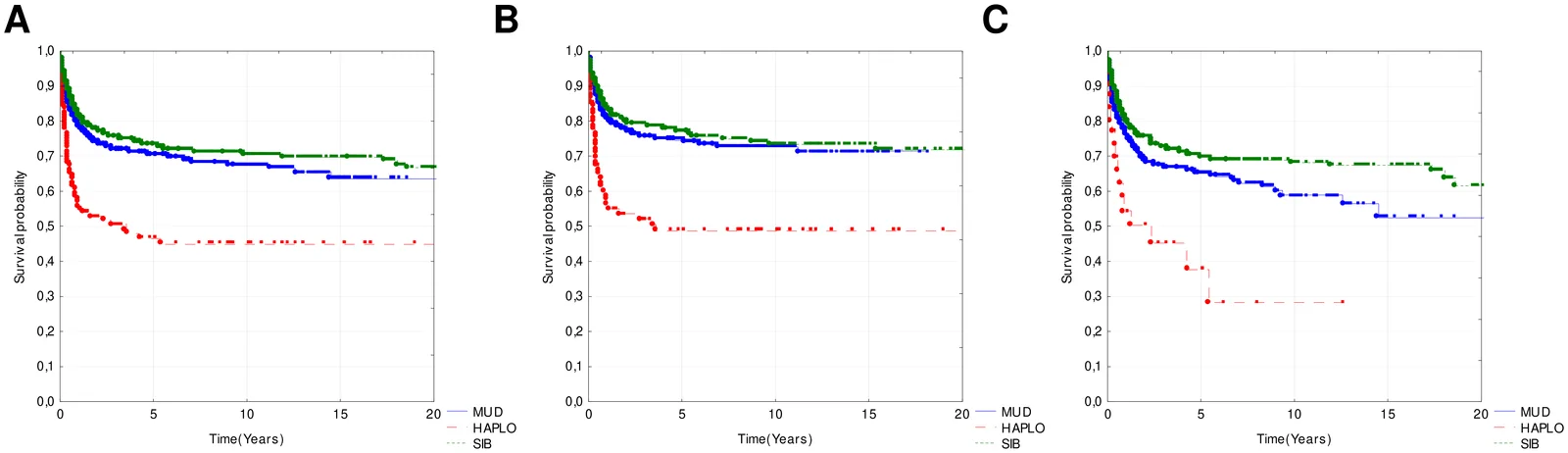
Overall survival after allo-HCT in children according to type of donor (MSD=SIB, UD, haplo): **(A)** total; **(B)** age <10 years; **(C)** age >10 years.

Children <10 years. Overall survival for children aged <10 years after allo-HCT (n=1207) with respect to donor type: for MSD-HCT (n=336) was 77.1% at 5 years and 73.4% at 10 years; for UD-HCT (n=781) was 74.5% at 5 years and 72.6% at 10 years; for haplo-HCT (n=90) was 48.6% at 5 years and 48.6% at 10 years (p<0.00001) (**Figure 6B**).

Children >10 years. Overall survival for children aged >10 years after allo-HCT (n=973) with respect to donor type: for MSD-HCT (n=380) was 69.7% at 5 years and 68.0% at 10 years; for UD-HCT (n=562) was 65.1% at 5 years and 58.9% at 10 years; for haplo-HCT (n=31) was 37.7% at 5 years and 28.2% at 10 years (p=0.00140) (**Figure 6C**).

### Outcome after allo-HCT (MSD, UD, HAPLO) in adults

Adults. Overall survival for all adults after allo-HCT (n=7288) with respect to donor type: for MSD-HCT (n=2597) was 55.3% at 5 years and 49.5% at 10 years; for UD-HCT (n=4244) was 56.0% at 5 years and 49.6% at 10 years; for haplo-HCT (n=447) was 44.8% at 5 years and 38.8% at 10 years (p=0.00030) (**Figure 7A**).

**Figure 7:**
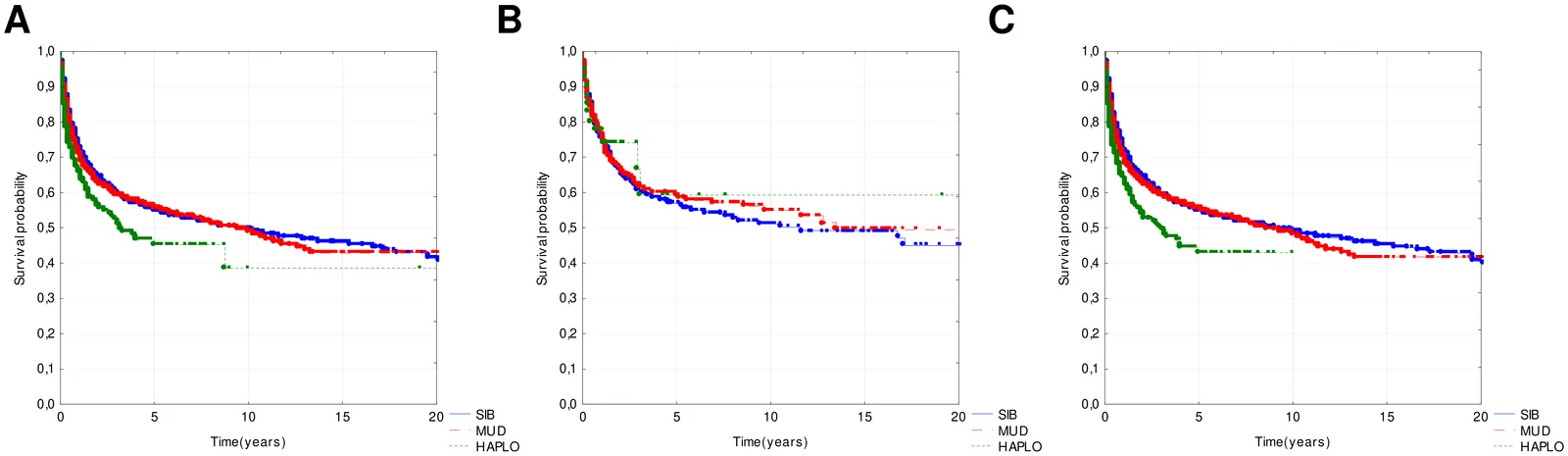
Overall survival after allo-HCT in adults according to type of donor (MSD=SIB, UD, haplo): **(A)** total; **(B)** age 18-25 years; **(C)** age >25 years.

Adults 18-25 years. Overall survival for adults aged 18-25 years after allo-HCT (n=791) with respect to donor type: for MSD-HCT (n=335) was 56.9% at 5 years and 51.2% at 10 years; for UD-HCT (n=414) was 60.0% at 5 years and 55.0% at 10 years; for haplo-HCT (n=42) was 59.1% at 5 years and 59.1% at 10 years (p=0.93645) (**Figure 7B**).

Adults >25 years. Overall survival for adults aged >25 years after allo-HCT (n=6497) with respect to donor type: for MSD-HCT (n=2262) was 55.1% at 5 years and 49.3% at 10 years; for UD-HCT (n=3830) was 55.6% at 5 years and 48.6% at 10 years; for haplo-HCT (n=405) was 43.2% at 5 years and 43.2% at 10 years (p=0.00008) (**Figure 7C**).

### Outcome after HCT in time periods in children

Children. Overall survival for all children after (allo+auto) HCT (n=3038) with respect to time period: before 2001 (n=255) was 51.8% at 5 years and 49.9% at 10 years; between 2001-2010 (n=1026) was 56.8% at 5 years and 53.2% at 10 years; after 2010 (n=1757) was 75.0% at 5 years and 68.0% at 10 years (p<0.00001) (**Figure 8A**).

**Figure 8:**
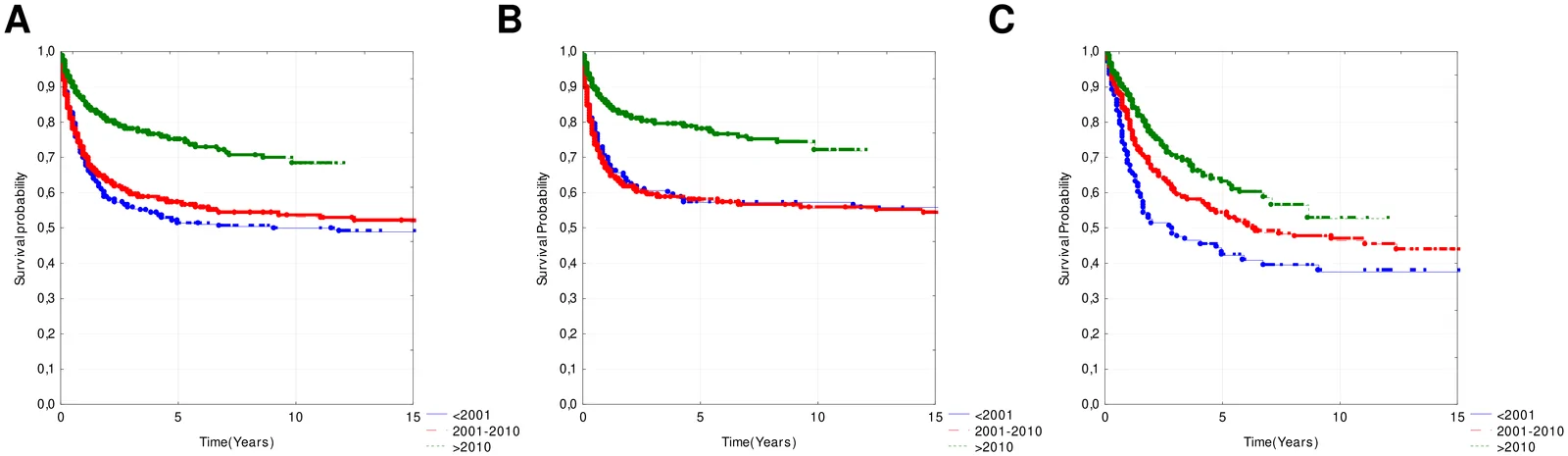
Overall survival after HCT in children according to time period (<2001; 2001-2010; >2010): **(A)** total allo+auto; **(B)** allo-HCT; **(C)** auto-HCT.

Children after allo-HCT. Overall survival for all children after allo-HCT (n=2180) with respect to time period: before 2001 (n=156) was 57.2% at 5 years and 57.2% at 10 years; between 2001-2010 (n=709) was 57.7% at 5 years and 55.6% at 10 years; after 2010 (n=1315) was 78.1% at 5 years and 72.1% at 10 years (p<0.00001) (**Figure 8B**).

Children after auto-HCT. Overall survival for all children after auto-HCT (n=858) with respect to time period: before 2001 (n=99) was 43.3% at 5 years and 37.5% at 10 years; between 2001-2010 (n=317) was 54.1% at 5 years and 46.4% at 10 years; after 2010 (n=442) was 63.6% at 5 years and 52.7% at 10 years (p<0.0001) (**Figure 8C**).

### Outcome after HCT in time periods in adults

Adults. Overall survival for all adults after (allo+auto) HCT (n=16861) with respect to time period: before 2001 (n=809) was 48.1% at 5 years and 43.9% at 10 years; between 2001-2010 (n=3604) was 59.1% at 5 years and 50.7% at 10 years; after 2010 (n=12448) was 69.3% at 5 years and 62.3% at 10 years (p<0.00001) (**Figure 9A**).

**Figure 9:**
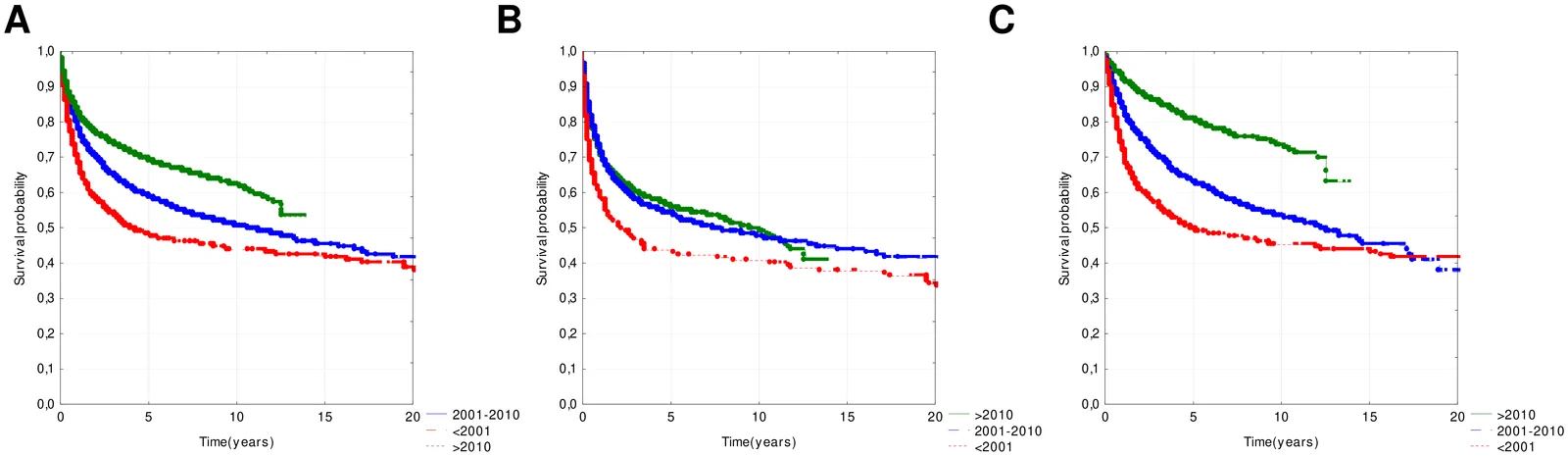
Overall survival after HCT in adults according to time period (<2001; 2001-2010; >2010): **(A)** total allo+auto; **(B)** allo-HCT; **(C)** auto-HCT.

Adults after allo-HCT. Overall survival for all adults after allo-HCT (n=7288) with respect to time period: before 2001 (n=218) was 43.5% at 5 years and 40.7% at 10 years; between 2001-2010 (n=1566) was 54.0% at 5 years and 47.7% at 10 years; after 2010 (n=5504) was 56.2% at 5 years and 49.6% at 10 years (p=0.00002) (**Figure 9B**).

Adults after auto-HCT. Overall survival for all adults after auto-HCT (n=9573) with respect to time period: before 2001 (n=591) was 49.8% at 5 years and 44.9% at 10 years; between 2001-2010 (n=2038) was 63.3% at 5 years and 53.2% at 10 years; after 2010 (n=6944) was 80.6% at 5 years and 73.3% at 10 years (p<0.00001) (**Figure 9C**).

## Discussion

Almost seven decades have passed since the first successful HCTs were performed in the America and Europe (11, 12). Since then, advances in genetics, immunology, and hematological therapies, as well as in the management of infectious and non-infectious complications, have transformed HCT from an experimental procedure into a standard treatment method.

This nationwide analysis summarizes four decades of HCT activity in Poland. Over the analyzed period, more than 30,000 HCTs were performed. During this time, the scope of transplant activity, donor selection criteria, indications, and patient characteristics underwent significant changes. The key finding was the observed improvement in survival rates over the years. The five-year survival rate rose from 57.2% before year 2001 up to 78.1% after 2010 among pediatric patients, and from 48.1% to 69% among adults. This improvement was recorded despite the increasing frequency of HCTs performed on older patients and those with more complex clinical profiles. An increase in the importance of unrelated and haploidentical donors, as well as changes in indications for transplantation, were also observed. These results illustrate the development of the HCT field in Poland and align with major trends observed across Europe.

The first successful HCT in Poland was performed in 1984, during a difficult period post martial law for the country. Due to limited opportunities for international collaboration, the bone marrow transplantation program was developed primarily based on available reports and scientific publications (8). These early successes paved the way for the further development of transplantation techniques, including stem cell mobilization and storage, conditioning regimens, and immunosuppressive therapy. In 1997, the first unrelated-donor HCT was performed in Poland. In subsequent years, the number of allogeneic transplantations exceeded 100 procedures annually. The development of donor registries improved donor availability (9). The establishment of the Polish donor registry in the early 2000s was of particular importance, as it improved access to unrelated donors (13, 14). This led to an increase in the number of allo-HCTs, particularly those involving unrelated donors. Similar trends were also observed in other European countries.

The introduction of haploidentical transplantation, particularly using post-transplant cyclophosphamide, has further increased donor availability for patients for whom no suitable matched donor was found. According to EBMT registry data, matched related donors and haploidentical donors account for approximately 25% and 19%, respectively, of allo-HCT cases (15). In our analysis, haploidentical transplants constituted 7.3% of all allo-HCTs in children and 6.5% in adults. Although these rates are lower than those reported by the EBMT, an increase in the number of haploidentical donor transplants has been observed in Poland in recent years, including patients requiring urgent intervention (16, 17).

Transplant programs in Central and Eastern Europe developed later than in Western Europe. However, countries such as Poland gradually increased their transplantation activity. While transplant rates in many Western European countries reached a plateau, activity in Central and Eastern Europe continued to rise, narrowing the gap between the two regions (5, 14). The increase in transplant activity observed in our study is therefore consistent with European trends. Advances in conditioning regimens, stem cell mobilization, cell processing, donor selection, and immunosuppressive therapy have contributed to the development of HCT and improved patient outcomes (18).

A significant finding of our study is the observed improvement in survival over time. Similar improvements in outcomes have been reported by other centers worldwide; this may be related to advances in donor selection, conditioning strategies, supportive care, infection control, and the management of transplant-related complications (1–3, 19). However, it should be noted that our survival analyses did not account for risk factors; consequently, the results should be interpreted as trends over time rather than as evidence of a direct impact of changes in transplantation practice. Differences regarding patient age, diagnosis, eligibility for the procedure, donor type, conditioning regimen, and supportive care may have contributed to the observed improvement.

Our study also highlights changes in indications for HCT over time. Myeloid neoplasms remain the most common indication for HCT in adults, consistent with European data (15, 18). However, certain indications have undergone significant changes. Chronic myeloid leukemia, previously a common indication for allogeneic HCT, is now less frequent following the introduction of effective targeted therapies. At the same time, the scope of indications for autologous HCT has expanded to include selected solid tumors and autoimmune diseases (20, 21).

The increasing availability of cell therapies, particularly CAR-T cell therapy, may further alter the indications for HCT (15). The role of autologous HCT may change, especially in the context of multiple myeloma and non-Hodgkin lymphoma (22). Indications for transplantation in pediatric patients may also evolve. Acute lymphoblastic leukemia currently represents a significant indication for allogeneic HCT; however, the role of this procedure may shift with the expanding use of CAR-T cell therapy and other novel treatments. Similar changes may also apply to adult patients. It should be noted, however, that our analysis covers the period prior to the widespread adoption of CAR-T cell therapy in Poland; consequently, it does not allow for an assessment of the therapy’s impact on the number of transplants performed.

In Poland, access to HCT has not been significantly constrained by financial factors since the early 2000s. Initially, a primary limiting factor was the relatively small number of transplant centers. Over time, indications for transplantation and the upper age limits for patient eligibility have been expanded. The use of reduced-intensity and non-myeloablative conditioning regimens has also enabled transplantation in older patients and individuals with significant comorbidities (23).

This study has several limitations. Due to the long period covered by the analysis, some data from earlier years are incomplete, and the dataset itself is heterogeneous. The follow-up also varied across the different study periods. Survival analyses were conducted without adjusting for confounding factors; therefore, they should be interpreted as reflecting trends over time. The results may have been influenced by changes in patient characteristics, diagnostic classification, HLA typing, donor selection, conditioning regimens, and supportive care. The interpretation of transplant activity and survival rates may also be affected by cases of re-transplantation. However, it should be noted that transplant procedures are mandatorily reported to Poltransplant, the organization maintaining the national transplant registry, which limits the risk of incomplete data capture. Data regarding disease relapse, transplant-related mortality, non-relapse mortality, graft failure, and early and late post-transplant complications were also incomplete, particularly for the earlier years. Consequently, a reliable analysis of these parameters was not possible, limiting the ability to interpret the factors contributing to improved survival.

Four decades of HCT in Poland illustrate the evolution from pioneering procedures to modern transplant practice aligned with international standards. The increase in transplant activity, changes in donor selection and indications, and improved survival rates reflect the progress achieved during this period. Future developments in the field will likely be shaped by advances in cell therapies, haploidentical transplantation, immunotherapy, and CAR-T cell therapy.

## Conclusions

This nationwide analysis demonstrates the significant development of HCT in Poland over the past four decades. A marked increase in transplantation activity has been observed, accompanied by changes in donor selection, indications for transplantation, and patient characteristics, as well as improvements in survival rates. These results reflect the evolution of HCT in Poland and align with major trends observed across Europe.

## Data Availability

The raw data supporting the conclusions of this article will be made available by the authors, without undue reservation.
